# Two Solutions of Soil Moisture Sensing with RFID for Landslide Monitoring

**DOI:** 10.3390/s18020452

**Published:** 2018-02-03

**Authors:** Sérgio Francisco Pichorim, Nathan J. Gomes, John C. Batchelor

**Affiliations:** 1 Graduate School of Electrical Engineering and Computer Science (CPGEI-DAELN), Federal University of Technology, Paraná (UTFPR), 80230-901 Curitiba, Brazil; 2 School of Engineering and Digital Arts, University of Kent, CT2 7NT Canterbury, UK; N.J.Gomes@kent.ac.uk (N.J.G.); J.C.Batchelor@kent.ac.uk (J.C.B.)

**Keywords:** soil, moisture, sensor, landslide

## Abstract

Two solutions for UHF RFID tags for soil moisture sensing were designed and are described in this paper. In the first, two conventional tags (standard transponders) are employed: one, placed close to the soil surface, is the sensor tag, while the other, separated from the soil, is the reference for system calibration. By transmission power ramps, the tag’s turn-on power levels are measured and correlated with soil condition (dry or wet). In the second solution, the SL900A chip, which supports up to two external sensors and an internal temperature sensor, is used. An interdigital capacitive sensor was connected to the transponder chip and used for soil moisture measurement. In a novel design for an UHF RFID tag the sensor is placed below the soil surface, while the transponder and antenna are above the soil to improve communication. Both solutions are evaluated practically and results show the presence of water in soil can be remotely detected allowing for their application in landslide monitoring.

## 1. Introduction

In Brazil, due to serious social problems, human occupation occurs in hillsides usually evaluated as risk areas. This exposes people to many hazards, especially landslide risks. In summer, tropical rains soak the soil of hillsides, causing tragedies. Unfortunately, landslides have repeatedly occurred in the last decade. Landslide disasters are related to several factors and natural events, such as earthquakes, volcanic activity, and, in particular, the degree of rain and soil water saturation. The ability to monitor the soil moisture, at many points of an urban slope where there are risk areas, could be a very important way of evaluating the degree of danger and, therefore, predicting and/or preventing accidents and deaths. Some studies have shown a clear and direct relationship between rainfall, soil moisture and landslide events [1,2]. Sensing networks and telemetric instrumentation have been applied to monitor soil moisture levels for landslide forecasting [1,2,3].

Ultra-high-frequency radio-frequency identification (UHF RFID) devices may be an interesting solution for soil moisture measurement because the sensors are low-cost, can be passive (no battery) and can provide communication distances of some meters [4,5]. A set of RFID-based soil moisture sensors properly installed on a hillside could provide real-time landslide monitoring.

Moisture level or liquid identification have been frequent applications of UHF RFID tags in the literature [6,7,8,9,10,11,12,13,14,15], for example, monitoring the beverage volume in cup or bottle [11,12], water in concrete [5], moisture in wall [13] or in soil [9,10,14,15]. The electrical properties of a tag can be modified by the presence of water in its proximity, causing, for example, changes or shifts in resonance frequency, the backscattered signal, or the activation energy (tag’s turn-on power) [11,12,14,15,16]. Different approaches can be found using cheap and conventional tags (i.e., standard non-sensing transponders) [8,11,12,13] or proposing new designs, where special probes connect the tag to the medium under measurement [5,14,15]. For system calibration, an interesting solution is the use of two tags, one affected by moisture and the other (as a reference) protected against moisture [6,13]. By frequency sweeping [6] or reader power ramping [17], the responses of tags can be measured and differences between them can be correlated to humidity level. 

This paper presents two solutions for the use of UHF passive RFID tags in soil moisture measurement, targeting particularly landslide monitoring.

## 2. Two Methods of Measurement 

For the use of UHF RFID tag as a soil moisture monitor, two solutions were proposed and designed. For the first approach, two conventional tags (adhesive labels) are placed in the same spot, where one works as an analog sensor and other as a reference. The sensitive tag (sensor) has its antenna characteristics modulated by soil moisture. This will change the threshold of its activation. The other tag (reference), which is made moisture insensitive, is used as a distance or power reference. The reader works at a constant frequency but with power sweeping (ramp). The difference between the responses of the tags can be used to determine soil moisture. Many UHF chip tags can be applied in this solution. For example, the G2XL is a chip for passive, intelligent tags and labels. Although it does not support any external sensor, it can be used as a tag sensor by changing the antenna tuning, impedance matching, or signal level [4,11,12,17]. In this case, the sensing is achieved through capacitive coupling arising from the antenna proximity to the soil surface.

For the second approach, a dedicated tag chip with external sensor input is used. There are few UHF chip tags available with sensor input, however some solutions are possible. For example, the chip G2iL+ is a 4-pin chip, allowing the tamper alarm application [18]; this 1-bit input (on or off) could be used as a digital sensor for soil moisture. For this purpose, a threshold between dry and wet soil must be triggered by a specific circuit.

A better solution for the second approach is the chip SL900A, which is an EPC global Class 3 sensor tag chip with automatic data logging [19]. It works with an internal temperature sensor and can read two external analog sensors [20,21,22,23], including resistive, capacitive and optic sensors. This solution will be developed and described further in this paper. 

## 3. First Approach: Two Conventional Tags

Initially, a test using a conventional UHF RFID tag (FT-G1210 RFID Inlays, NXP, Eindhoven, Netherlands) on dry and wet soil was conducted to understand the influence of soil moisture on the tag’s power on threshold. The influence was evaluated at different separation distances (*H*) between the tag and soil surface.

**Figure 1:**
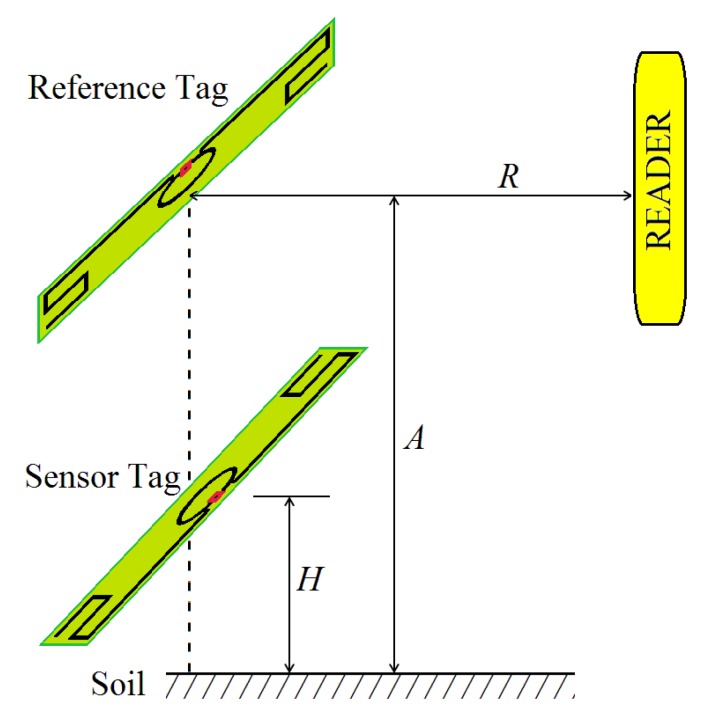
System using two adhesive UHF RFID tags for soil moisture monitoring. Sensor and reference tags, with distance *H* and *A*, respectively, from soil. The reading distance is *R*.

The soil moisture sensor tag is placed close to the ground (Figure 1) at height *H*. The highest sensitivity of the tag was found to be when *H* was a few millimeters, as will be described below. A second tag, which is used as a reference by the reader (distance *R*), is separated from the soil with height *A*; it must be placed far from the soil (in air), some centimeters high, in order to be insensitive to soil moisture. Both tags are parallel to the soil but perpendicular to each other, as seen in Figure 1. 

The behavior of the tags was measured by a Tagformance Lite Reader (Voyantic, Espoo, Finland) in order to determine the best height *H*. This equipment applies ramps of power and analyses the response of a tag population. Thus, the transmission power necessary to turn on the tags (*P_TagOn_*) can be evaluated. Frequencies between 800 MHz and 1 GHz were tested.

The setup shown in Figure 1 was placed on dry and wet soils and the Tagformance Lite Reader’s antenna was oriented to avoid signal reflections from the soil. Initially, the distance between reader antenna and tag (*R*) was set to 7 cm.

The test was conducted using 2200 cm^3^ of topsoil (black earth collected in coordinates 51.297254 North and 1.063208 East), which was dried before the tests. After the first tests (for dry soil situation), a volume of 500 cm^3^ of tap water was added to the soil. Then, the wet soil tests were started some minutes later, after the water had completely percolated. This volume of water represents a humidity of 22.7% (volume ratio), which can be considered a very wet soil (soaked). For black earth with bulk density of 1.1 g/cm^3^, this volume ratio represents a weight ratio of 20.7% of water in soil. It is a level that can be assumed within a risk range for a landslide event [2].

**Figure 2:**
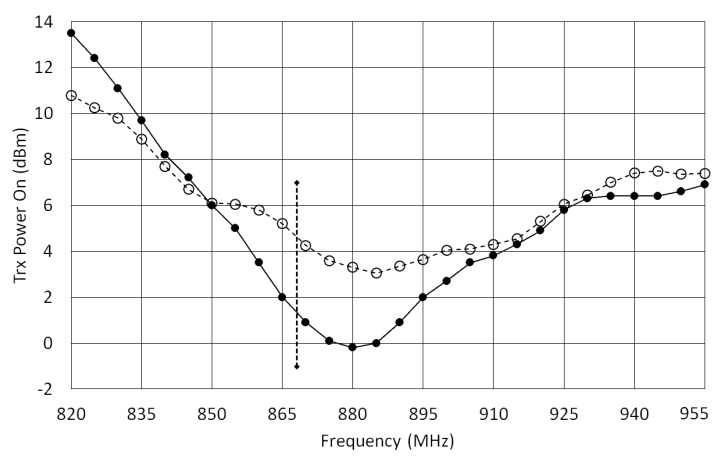
Transmission power to turn on the tag (for *H* = 26 mm) as a function of frequency. Solid line is for wet soil and dashed line is for dry soil. The cursor marks the frequency of 868 MHz.

In Figure 2 the behavior of a sensor tag over dry and wet soil (dashed and solid lines, respectively) was observed for a tag height (*H*) of 26 mm as a function of the reader’s frequency. The presence of water in the soil modifies the power necessary to turn on the tag, especially around 870 MHz. For the European UHF RFID frequency (868 MHz, marked with a cursor in Figure 2), the turn-on power (*P_TagOn_*) changes from about 1.5 to 5 dBm as a function of soil humidity. This significant variation can be exploited by a soil moisture monitoring system. Analyzing the results of Figure 2, it is possible to see that, at 868 MHz, the tag is more sensitive to soil moisture (dry and wet) than at 915 MHz. Of course, other tag models, or other antenna designs, may exhibit different behavior.

**Figure 3:**
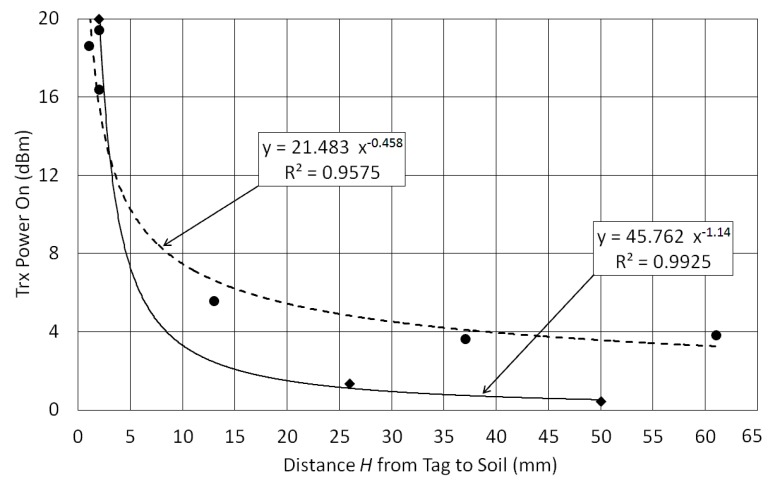
Transmission power to turn on the tag (for 868 MHz) as a function of the distance between tag and soil (*H*). Diamonds (solid line) are for wet soil and dots (dashed line) are for dry soil.

Adopting the frequency of 868 MHz, a second test was made to evaluate the tag’s behavior as a function of the height *H*, with measurements obtained for *H* from 1 to 60 mm over both dry and wet soil, as shown in Figure 3. When the tag is very close to the soil (*H* lower than 5 mm) the transmission power *P_TagOn_* is greater than 12 dBm for both soil situation (dry and wet), thus the tag becomes insensitive. Of course, for greater heights the sensor tag will be also insensitive. A maximum sensitivity is obtained when the tag is placed 10 to 30 mm above the soil, as can be seen in Figure 3.

**Figure 4:**
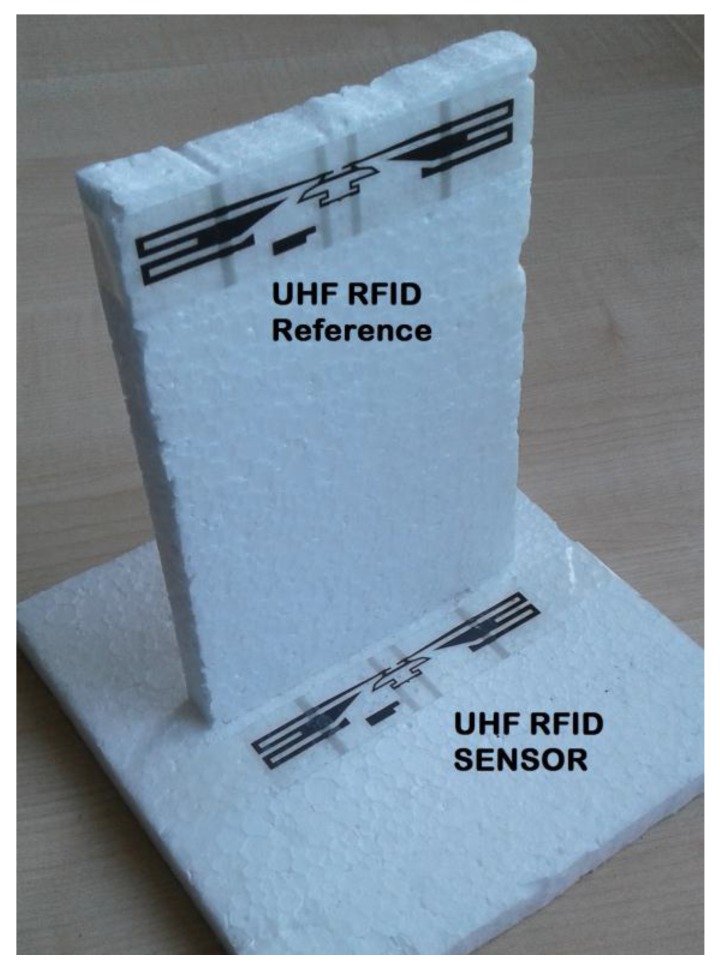
Prototype of Soil Moisture Sensor using two adhesive UHF RFID tags. The Sensor tag is place 12 mm over the soil. A Reference tag is 100 mm apart from the soil.

**Figure 5:**
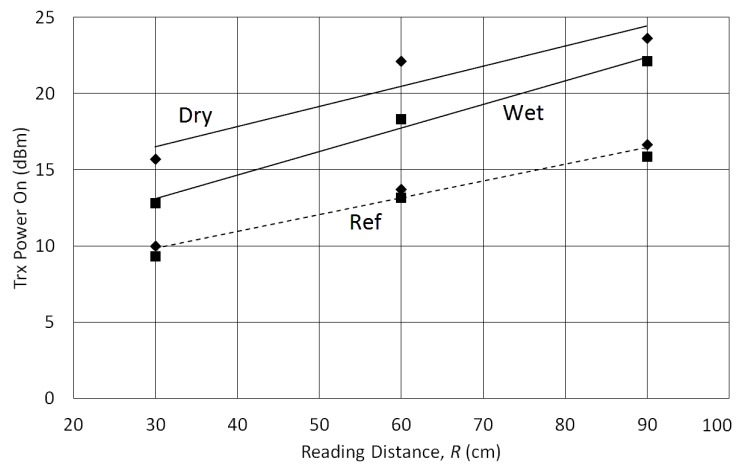
Transmission power to turn on the tags (for 868 MHz) as a function of the reading distance between tags and reader (*R*). Solid lines are for Sensor Tag and dashed line for Reference Tag. Diamonds are for dry soil and squares are for wet soil.

Using the results of Figure 3, a system with two conventional UHF RFID tags (adhesive labels) was designed. The sensor tag was fixed with *H* = 12 mm and a second tag (the reference one) was placed with *A* = 100 mm, where the tag was assumed to be insensitive to soil moisture. A photo in Figure 4 shows how these tags are attached. This prototype, only for indoor evaluation, was constructed with a structure of expanded polystyrene blocks, which is assumed to be an UHF neutral material. The reading distances (*R*) of 30, 60 and 90 cm were used to measure the transmission power *P_TagOn_* for sensor and reference tags over dry and wet soils, as seen in Figure 5.

According to test results with the Tagformance Lite Reader, a theoretical read range *R* up to 4 m can be expected. There is a difference of about 7 to 8 dB between sensor and reference tags when the soil surface is dry. However, this difference decreases to about 4 to 5 dB when the soil is wet. These differences are relatively constant for variations of distance *R*.

It can be noticed that the reference sensor (dashed lines) is almost completely insensitive to soil moisture. On the other hand, a significant variation is observed for sensor tag response. Therefore, this behavior can be exploited as a system to monitor soil moisture using two conventional tags.

## 4. Second Approach: Using SL900A Chip

**Figure 6:**
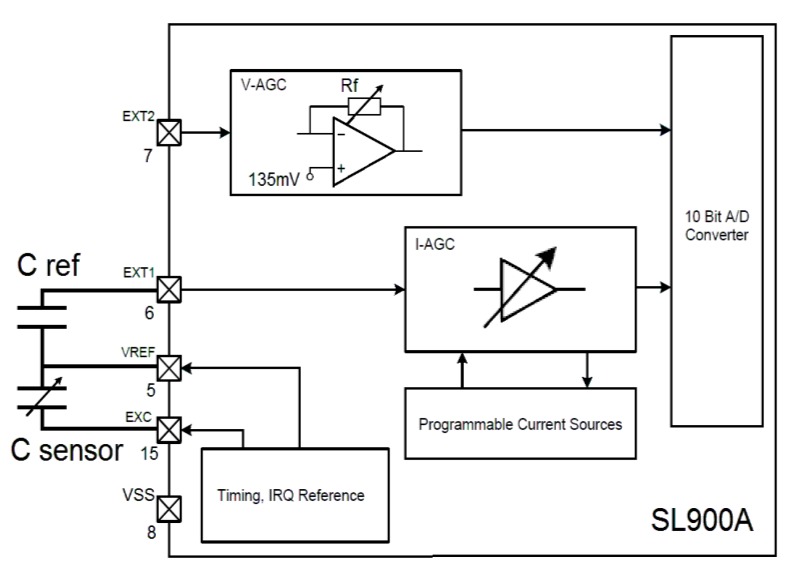
Block diagram of External Sensor Interface of SL900A. For a capacitive sensor (*C sensor*) a reference capacitor (*C ref*) must be inserted in Ext1 pin.

The SL900A (AMS AG, Premstätten, Austria) is an UHF RFID chip with an internal temperature sensor and two other inputs for external analog sensors. These inputs can support many types of sensor, such as, resistive, conductive, capacitive, current, voltage, resistive bridge and photodiode [19]. The Sensor Front End (SFE) of the SL900A is composed of an integrated 10-bit Analog to Digital Converter (ADC) with selectable voltage references (Vo1 and Vo2). In addition, a current source is used for sensor excitation (data field “Seti”). An operational amplifier feedback resistance (data field “Rang”) and the signal frequency (data field “Df”) can be configured in the SFE (see Figure 6). The frequency of the RTC oscillator can be calibrated from 800 to 1165 Hz. The references of the ADC are selected for *Vo1* (160 to 510 mV) and *Vo2* (260 to 610 mV). These voltage references are individually selectable in 50 mV steps [19]. For a capacitive sensor, the circuit is connected as shown in Figure 6. 

The SL900A chip uses commands EPC (Gen2) and cool-Log^®^ for its configuration, communication and setting. These commands are transmitted by the tag’s reader using a Graphic User Interface (GUI) program. A reader kit DK-R902 LP2 (IDS Microchip, Wollerau, Switzerland) and its GUI, R90xG Demonstrator Control (version 2.12), was used to configure and read the developed tag sensor. The basic commands, used for configuring the chip’s SFE interface and reading the chip’s ADC output, are:

- Get measurement setup (bytes 0xE0 and 0xA3). Tag replies 128 bits with setup information;
- Get calibration data (bytes 0xE0 and 0xA9). Tag replies 72 bits with current calibration;
- Set SFE parameters (bytes 0xE0 and 0xA4). Reader sends 16 bits for sensor front end setup;
- Set calibration data (bytes 0xE0 and 0xA5). Reader sends 56 bits for tag’s calibration; and
- Get sensor value (0xE0 and 0xAD). Tag answers 6 bits of status and 10 bits of ADC value.

Using the above commands from the GUI software, the sensor front end of SL900A was appropriately configured.

The complete circuit for this second approach is shown in Figure 6, where the SL900A is configured for a capacitive sensor in Ext1. Initially, a simulation of capacitive sensor input was performed, in order to test the communication, configuration and response of the circuit and chip. Some capacitors were connected to yield a range from 15 to 88 pF. Five readouts, obtained by the GUI and reader, were collected for each input capacitance. The reference capacitor (*C ref*) of 27 pF was determined in function of ADC voltage limits and the range of a real moisture sensor’s capacitance. A correlation of 0.9969 between input capacitances and the GUI readouts was calculated, with an RMS error of 5.15%. When the input capacitance is low, the readouts are more unstable, probably, due to stray capacitances of wires, or even noise.

**Figure 7:**
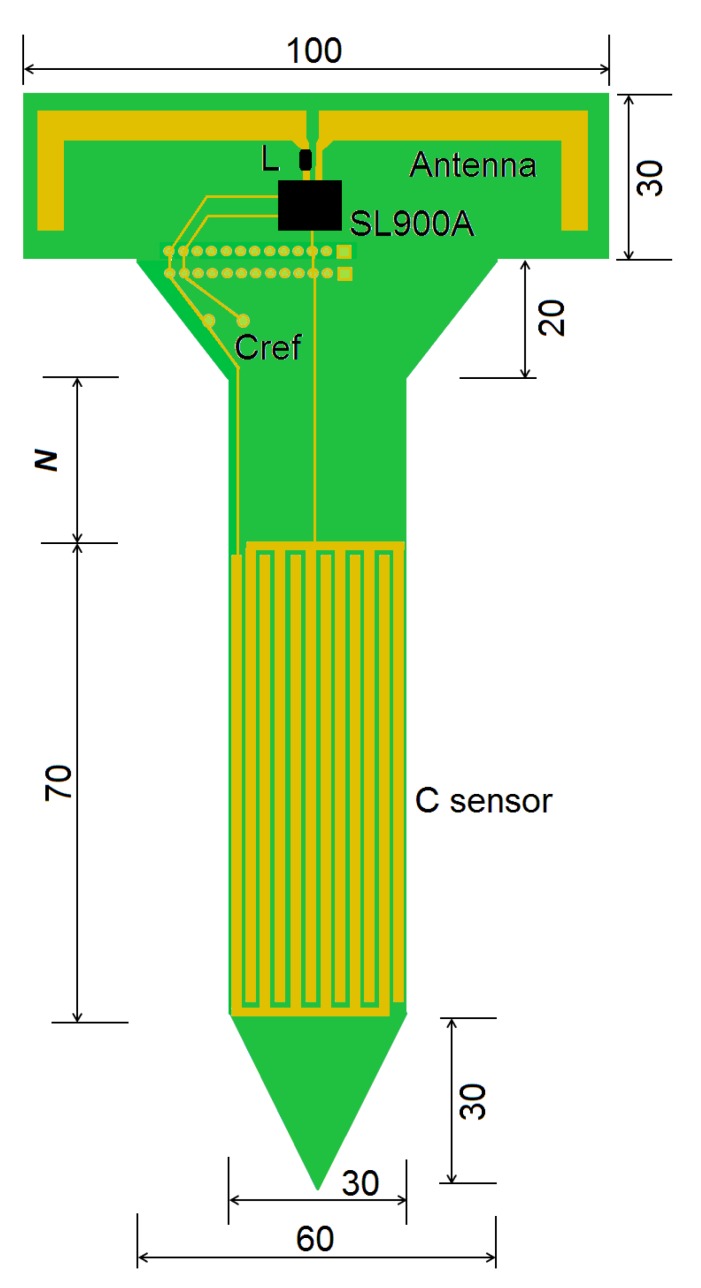
Design of the Soil Stick Sensor using UHF RFID chip (SL900A) and an interdigital capacitive humidity sensor (*C sensor*). A neck of *N* = 30 mm was defined. The arrow shape allows the tag to be stuck into soil. The whole sensor area must be buried. All dimensions are in millimeter (mm).

To avoid this instability, an interdigital sensor was designed to work at higher capacitances (ranging to about hundreds of picofarad). The capacitive sensor for the soil moisture measurement is a set of 12 interdigital tracks of copper constructed directly on the printed circuit board (PCB) of the UHF RFID tag, as shown in Figure 7. The sensor area (70 × 30 mm^2^) is covered with PCB ink for protection against corrosion. The tracks are designed with 2 mm of width and gaps of 0.5 mm between them. 

The sensor’s range of capacitance variation depends on the PCB substrate and ink permittivity, as well soil characteristics and moisture level. In practice, the total sensor capacitance varied between about 70 to 700 pF, for dry and wet soil, respectively. Thus, a reference capacitor (*C ref*) of 220 pF was chosen and inserted between the pins 5 and 6 of chip SL900A.

In this work, the RFID tag sensor is a passive device (no battery), although the SL900A can also work actively with a 3-V coin battery. The tag’s antenna is a dipole (L shape) with an SMD inductor (39 nH) for impedance matching [20,24]. The total height of the tag can be increased. The tag’s neck (length *N* in Figure 7) can be defined or changed as a function of how deep the sensor area (*C sensor*) must be inserted into the soil while the tag’s antenna is elevated from the soil, aiming towards communication range improvement.

**Figure 8:**
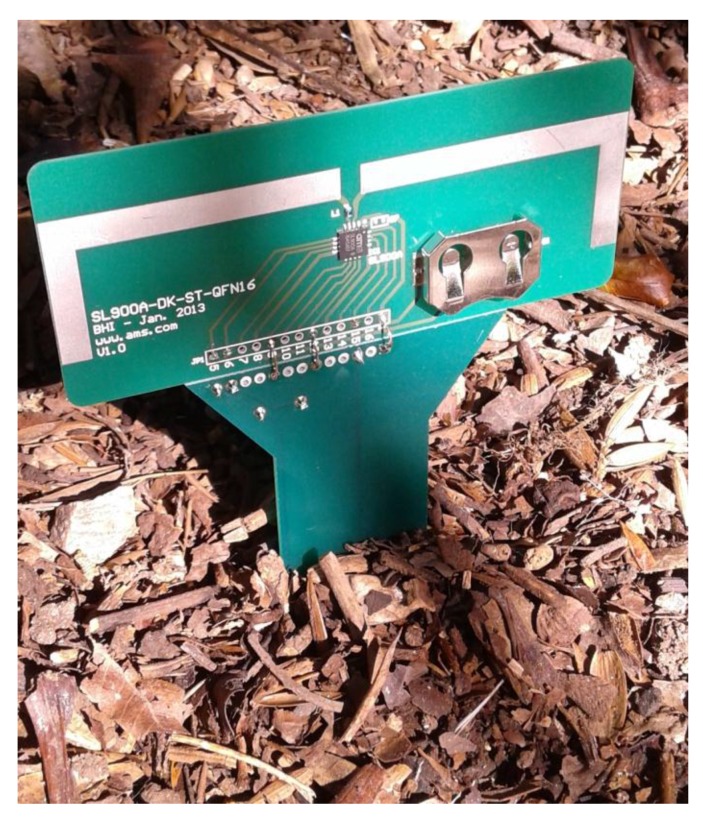
Example of the Soil Stick Sensor for Humidity in a real situation.

A new test was performed with the complete tag to analyze the system performance in soil with different moisture levels. The UHF RFID tag (Figure 7) was stuck into a soil sample in the laboratory (as can be seen in Figure 8). The soil moisture was estimated by the weight ratio of moist and dried soil, using a digital scale (resolution of 0.1 g). For this test, the topsoil (volume of 650 cm^3^) was collected from coordinates 25.533168 South and 49.200655 West.

**Figure 9:**
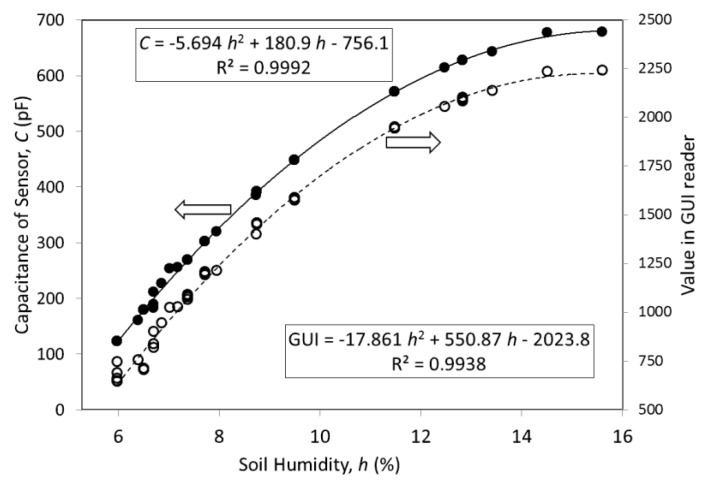
Sensor capacitance (solid dots and line) as a function of soil moisture *h* (weight ratio). Dashed line and hollow dots are values obtained by GUI of UHF RFID reader. Curves are parabolic approximations.

The soil was wetted (140 cm^3^ of tap water) and the moisture level was monitored during consecutive days, while it was drying naturally. The sensor capacitance was measured by an impedance analyzer (model 4294A, Agilent, Santa Clara, CA, USA). Figure 9 shows the relationship between soil moisture (weight ratio, *h*) and the sensor capacitance, where a nonlinear behavior is observed. A simple approximation, using parabolic curve fitting, was applied to describe its tendency. The plotted curve presented a correlation of 0.9992.

The sensor capacitance (*C*) can be read by the tag’s IC (SL900A) and by the UHF reader’s GUI software, as shown on the right axis of Figure 9. 

For a very dry soil (*h* smaller than 6%), the sensor capacitance tends to a saturation value of about 100 pF. When the soil is soaked (*h* greater than 16%), the capacitance becomes saturated at about 680 pF.

## 5. Discussion

The prevention of disasters involving landslides, especially those caused by excess water or moisture in the soil is the major motivation of this research. In this paper two solutions were presented to monitor soil moisture through RFID technology, especially using UHF tags. The novelty presented in this paper is the use of two passive tag options (with and without built-in sensor) to solve the problem of landslide on slopes with human occupation. The main advantage of using RFID tags is the absence of batteries in the remote modules (RFID sensors) and the power supply is only in the reader modules, which may be installed together with public lighting posts, for example.

Two conventional UHF RFID tags can be utilized to monitor soil moisture. Significant differences in turn-on power levels of 7 dB and 5 dB between sensor tag and reference tag for dry and wet soils, respectively, were observed in practice. Although this first solution uses ultra-low-cost tags, the determination of soil moisture needs an UHF RFID reader with power sweep functionality, i.e., an ability to produce power ramps to determine *P_TagOn_*. A reader with this facility is a high-cost piece of equipment; therefore, the first solution presents some problems when used in an urban area.

For the second option, the use of a dedicated chip (SL900A), its reader kit and the software (GUI) delivered by the manufacturer, simplified technically the project. Because of the dedicated sensor interface and 10-bit analog-to-digital conversion, the best results were obtained. Moreover, the interdigital capacitive sensor allows the determination of soil moisture level with higher accuracy and resolution. Although the SL900A tag is more expensive than conventional tags, its reader is simpler and relatively low-cost, allowing an outdoor implementation. Finally, this last solution is interesting, from the research and scientific point of view, if a second external sensor is inserted in the UHF RFID tag, since this input (Ext2) is available with the SL900A. In this case, beyond temperature and soil moisture measurements, other landslide parameters, such as rain precipitation level, soil movement or displacement, etc., could be monitored.

Briefly, the main advantage of the first solution is the simplicity of the sensor, which uses just ordinary tags, however its disadvantage is the high cost of the reader module. On the other hand, the second solution has the advantage of low cost, although it needs the design of a dedicated tag with sensor for the function. Another advantage of the latter solution is the possibility of including other type of sensor, or even two sensors. These characteristics can guide the definition of which solution is better to be used.

The main advantage of both solutions presented in this paper is that the RFID sensors are for long-term applications, due to the sensors are passive, i.e., they work without internal battery. The use of internal battery, in a sensor network, for example, requires periodic interventions in the system for batteries’ replacement. 

Although there is a direct relation between soil moisture level and landslide risk, other geological and climatic parameters, such as, precipitation or rainfall [2], should be considered for landslide forecasting. However, soil moisture is one of the most important and frequently used as a first indication of landslide alert. The volume ratios of water in soil, used in these experiments, represent high levels that can be assumed within a risk range for a landslide event [1,2,3].

It is important to point out that the presented experiments have been tested in a sufficient number of times to demonstrate that both techniques are functional in practice. For the implementation in a real environment (urban slope), the tests must be remade and repeated in a greater number of times (including statistical analysis) to obtain the behavior curves of the tags and the sensor more accurate with the local soil. Thus, the final performance must be evaluated thoroughly. This is because, for both solutions, the system calibration has a strong dependence on the soil characteristics (composition, density, porosity, etc.) of the hillsides, which will be monitored. Of course, this procedure is beyond the scope of this paper.

For the passive RFID approach, the distances over which the sensors can be interrogated is limited to below few meters, assuming good, directive antenna design and matching. Thus, the location of the sensors must be known and the reader antennas should be placed on street furniture such as lamp-posts on the outskirts of the populated area to be protected against landslide. The availability of electrical power at the lamp-posts will enable relatively high power interrogating signals, too. Multiple antenna heads can be placed on one lamp-post, aimed at different sensors but connected to a single reader. In this scenario, the RFID readers would be interconnected by any telecommunications infrastructure, with data eventually hopped back via a gateway node, collecting the information from multiple readers, through the internet to a monitoring station.
